# Effect of Graft Development in PLLA-Functionalized Cellulose Nanocrystals on Dispersibility, Transparency, and Mechanical Reinforcement in PLA Nanocomposites

**DOI:** 10.3390/polym18192370

**Published:** 2026-09-29

**Authors:** Hee Cheol Kang, Jae Woo Chung

**Affiliations:** Department of Materials Science and Engineering, Soongsil University, 369 Sangdo-ro, Dongjak-gu, Seoul 06978, Republic of Korea; wkdehrgml@gmail.com

**Keywords:** cellulose nanocrystals, poly(lactic acid), surface modification, PLLA graft development, nanocomposites

## Abstract

Despite their potential as reinforcing agents for poly(lactic acid) (PLA), cellulose nanocrystals (CNCs) tend to self-aggregate, limiting both optical transparency and mechanical reinforcement. In this study, incorporating poly(L-lactic acid)-grafted CNCs (LCNCs) into PLA simultaneously enhanced both optical transparency and mechanical reinforcement compared to neat CNCs. Three types of LCNCs—LCNC1 (apparent molar substitution, MS_app_ = 3.36), LCNC2 (MS_app_ = 25.6), and LCNC3 (MS_app_ = 56.9)—were synthesized. Fourier transform infrared (FT-IR) and ^1^H nuclear magnetic resonance (^1^H NMR) spectra revealed poly(L-lactic acid) (PLLA) grafting onto the CNC surface, and the MS_app_ values were estimated from the ^1^H NMR peak integrals. X-ray diffraction patterns (XRD) showed that the native crystalline structure of the CNCs was preserved even after surface modification. LCNC3 (MS_app_ = 56.9) exhibited a smaller hydrodynamic size in an organic solvent, consistent with reduced aggregation, whereas LCNC1 (MS_app_ = 3.36) remained severely aggregated like neat CNCs. The PLA/LCNC3 nanocomposite retained transparency comparable to neat PLA and achieved the highest tensile strength among the nanocomposites considered here. These results indicate that a higher MS_app_, reflecting greater PLLA graft development on CNCs, is associated with improved dispersion, optical transparency, and mechanical reinforcement in PLA nanocomposites.

## 1. Introduction

Petroleum-based plastics are widely used in various industries because of their low cost and excellent mechanical properties. However, the accumulation of petroleum-based plastic waste in landfills and water bodies has become a critical environmental challenge, as microplastic contamination and ecosystem pollution have led to an urgent demand for sustainable alternatives [1,2]. Biobased polymer nanocomposites have gained popularity as viable replacements for petroleum-based plastics, with comparable mechanical performance as well as good biodegradability and renewability [3,4,5]. Biobased nanocomposites formed by the incorporation of cellulose nanocrystals (CNCs) into a poly(lactic acid) (PLA) matrix have gained considerable attention as renewable nanocomposite systems [6,7].

PLA is a hydrophobic, biodegradable thermoplastic derived from renewable plant resources such as cornstarch and sugarcane, and its mechanical properties are suitable for packaging, textile, and biomedical applications [8,9]. In contrast, CNCs are hydrophilic nanoparticles isolated from diverse biosources (wood, cotton, and agricultural residues) by acid hydrolysis. CNCs possess high tensile strength, low density, and good biocompatibility [10,11,12]. This inherent incompatibility between the hydrophobic PLA and the hydrophilic CNCs leads to poor dispersion, strong aggregation, and weak interfacial adhesion, degrading the optical transparency and reinforcing efficiency of the resulting PLA/CNC nanocomposites [13,14,15].

Surface modification of CNCs with polymers has been proven to be an effective strategy to overcome this challenge by reducing the surface hydrophilicity of CNCs and enhancing matrix compatibility [16,17]. In particular, functionalization of CNCs with matrix-identical polymers is especially effective in improving filler dispersion and interfacial adhesion, leading to substantial improvements in the properties of the composite [18,19]. For example, Peng et al. recently demonstrated that grafting long-chain poly(ε-caprolactone) (PCL) diols onto CNCs markedly improved their interfacial compatibility with the PCL matrix, as evidenced by a substantially reduced interfacial tension compared to unmodified CNCs [20]. Similarly, Wu et al. showed that lactic acid-grafted CNCs (CNC-g-LA) improved filler dispersion and interfacial compatibility, resulting in enhanced mechanical properties and an accelerated crystallization rate of PLA nanocomposites [21]. In a more directly relevant study, de Paula et al. demonstrated that poly(L-lactic acid)-grafted CNCs, unlike ungrafted CNCs, markedly influenced the crystallinity of poly(L-lactic acid) (PLLA) nanocomposites, thereby enhancing their mechanical properties [22]. Nevertheless, despite these advances in PLLA-grafted CNC systems, uniform filler dispersion, strong mechanical reinforcement, and enhanced optical transparency remain difficult to achieve simultaneously in PLA nanocomposites. In particular, the effect of the grafted polymer chain development on the dispersion, optical transparency, and mechanical properties of these nanocomposites is insufficiently understood.

In this study, three types of PLLA-grafted CNCs (LCNCs) with similar apparent degrees of substitution (DS_app_) but different degrees of polymerization (DP) and apparent molar substitution (MS_app_) values were synthesized by controlling the monomer (L-lactide) feed ratio. In this context, the term “PLLA graft development” refers to the overall progression of PLLA grafting, represented by MS_app_, rather than an independently measured parameter. Their effects on the dispersion, optical transparency, mechanical properties, and stress relaxation behavior of the resulting PLA nanocomposites were then investigated. Our results showed that progressive PLLA graft development, accompanied by increasing graft-chain DP, was associated with improved nanoparticle dispersion and, consequently, enhanced optical transparency and mechanical reinforcement of the nanocomposites. These findings demonstrate that the extent of PLLA graft development is an important design parameter for achieving both optical transparency and mechanical reinforcement in PLA nanocomposites.

## 2. Materials and Methods

### 2.1. Materials

PLA (NatureWorks™ Ingeo 4060D, specific gravity: 1.24 g cm^−3^, D-lactic acid content: 11–13%) was supplied by NatureWorks Inc. (Plymouth, MN, USA). CNC powder was purchased from CelluForce, Inc. (Montreal, QC, Canada). N,N-Dimethylformamide (DMF, anhydrous, 99.8%) was purchased from Sigma-Aldrich (Seoul, Republic of Korea). Dichloromethane (DCM, 99.8%) and chloroform (CHCl_3_, 99.8%) were obtained from Daejung Chemicals & Metals Co., Ltd. (Siheung-si, Gyeonggi-do, Republic of Korea). L-Lactide (98%) and 1,5,7-Triazabicyclo[4.4.0]dec-5-ene (TBD, 98%) were purchased from Tokyo Chemical Industry Co., Ltd. (Tokyo, Japan).

### 2.2. Preparation of Poly(L-Lactide)-Grafted Cellulose Nanocrystals (LCNCs)

The synthesis of the LCNC powder is shown in Figure 1. CNCs (1 g) were vacuum-dried at 90 °C for 24 h. The dried CNCs were dispersed in anhydrous DMF (40 mL) with ultrasonication (ULH-700N, Ulsso Hitech, Cheongju, Republic of Korea) at 40% power for 10 min, followed by the dissolution of L-lactide (5 g, 0.035 mol). The mixture was heated at 40 °C and stirred under a nitrogen atmosphere for 1 h to ensure homogeneous dispersion prior to polymerization. Then, the TBD catalyst (3.4 wt% of the monomer) was added, and the reaction was allowed to proceed for 2 h with mechanical stirring. The product was purified by repeated centrifugation (10,000 rpm, 4 cycles) using a chloroform/hexane cosolvent to remove any residual catalyst and homopolymer. The obtained products were redispersed in chloroform and spray-dried using a Büchi B-290 spray dryer (Büchi, Flawil, Switzerland) to yield a white powder of poly(L-lactide)-grafted CNCs, denoted as LCNC2. The control samples, LCNC1 (amount of L-lactide reduced to 1 g) and LCNC3 (amount of L-lactide increased to 10 g), were prepared in the same manner as LCNC2.

### 2.3. Preparation of PLA/LCNC Nanocomposite Films

PLA 4060D pellets were dried at 90 °C for 24 h before use. The dried PLA (5 g) was dissolved in DCM (200 mL) and stirred at room temperature for 2 h. The LCNC fillers (LCNC1, LCNC2, or LCNC3; 50 mg, 1 wt% PLA) were added and stirred for an additional 6 h to obtain a homogeneous PLA/filler solution. The solutions were cast onto glass dishes and dried at room temperature for 24 h. The dried films were chopped and vacuum-dried at 90 °C for 6 h, followed by hot-pressing at 130 °C under a pressure of 40 MPa for 9 min to fabricate PLA/LCNC nanocomposite films with a thickness of 0.5 mm. For comparison, PLA/CNC nanocomposite films with unmodified CNCs were prepared under identical conditions.

### 2.4. Characterization

Fourier transform infrared (FT-IR) spectroscopy (Vertex 70, Bruker, Ettlingen, Germany) was performed in attenuated total reflectance (ATR) mode, and the spectra were obtained in the wavenumber range of 4000–600 cm^−1^ with a spectral resolution of 4 cm^−1^. ^1^H nuclear magnetic resonance (^1^H NMR; ECZ500/S1, 500 MHz, JEOL Ltd., Akishima, Tokyo, Japan) spectra were obtained using samples dispersed in DMSO-d_6_ at a concentration of 5 mg mL^−1^. The spectra were acquired using 32 scans with a relaxation delay of 4 s. X-ray diffraction (XRD; D2 Phaser, Bruker AXS, Karlsruhe, Germany) patterns were collected using Ni-filtered Cu Kα radiation (λ = 0.154 nm) at 30 kV in the 2θ range of 5–40°. Differential scanning calorimetry (DSC; Discovery DSC25, TA Instruments, New Castle, DE, USA) was performed under a nitrogen atmosphere at a heating rate of 10 °C min^−1^. Samples were held at –90 °C for 5 min, heated to 180 °C to erase thermal history, cooled to –90 °C, and reheated to 180 °C. Thermogravimetric analysis (TGA; TGA/DSC 1, Mettler Toledo, Schwerzenbach, Switzerland) was performed under a nitrogen atmosphere from 35 to 400 °C at a heating rate of 10 °C min^−1^. Transmission electron microscopy (TEM; Talos F200S, Thermo Fisher Scientific, Waltham, MA, USA) was performed at 200 kV. The CNC and LCNC specimens were drop-cast onto carbon-coated Cu grids (CF200-Cu, 200 mesh). Dynamic light scattering (DLS; DLS-8000, Otsuka Electronics Co., Ltd., Hirakata, Osaka, Japan) was performed on CNC and LCNC specimens dispersed in DCM (0.05 mg mL^−1^). UV–vis spectroscopy (UV-3600, Shimadzu Corporation, Kyoto, Japan) was performed to evaluate the optical transparency of the CNC and PLA/LCNC nanocomposite films over the wavelength range of 400–800 nm at room temperature. The mechanical properties of the nanocomposite films were evaluated using a universal testing machine (UTM; TD-U01, Technology and Dorf Inc., Bucheon, Republic of Korea) equipped with a 200 N load cell according to ASTM D638 Type V [23] Tensile tests were conducted at a crosshead speed of 10 mm/min. For the tensile properties, at least five specimens were tested for each composition, and the results are reported as the mean ± standard deviation. Statistical analysis of the tensile properties was performed using one-way analysis of variance (ANOVA) followed by Tukey’s post hoc test, with statistical significance defined at *p* < 0.05. The tensile fracture surfaces of neat PLA, PLA/LCNC1, and PLA/LCNC3 were observed using field-emission scanning electron microscopy (FE-SEM; GeminiSEM 300, Carl ZEISS AG, Oberkochen, Germany) at an accelerating voltage of 1 kV. Dynamic mechanical analysis (DMA) was performed using a DMA 850 analyzer (TA Instruments, USA) in tension mode on samples with a thickness of 0.5 mm. Temperature sweep tests were performed from 40 to 120 °C at a heating rate of 3 °C/min and a frequency of 0.1 Hz. Stress relaxation tests were conducted by equilibrating the samples at 40 °C and then applying 0.1% strain for 500 s. The UV–vis, DLS, DSC, TGA, DMA, and stress-relaxation measurements were performed 2–3 times for each sample.

## 3. Results and Discussion

### 3.1. Chemical Structure of LCNCs

The LCNC series (LCNC1, LCNC2, and LCNC3) was synthesized via ring-opening polymerization (ROP) of L-lactide from the surface hydroxyl groups of the CNCs. For this synthesis, DMF was employed as the reaction medium to prevent CNC aggregation and achieve homogeneous surface modification. TBD, a strong organic base, was selected as the catalyst because of its superior activity in the ROP of the lactide moiety on cellulose as compared to conventional catalysts such as 4-dimethylaminopyridine (DMAP) or Tin(II) 2-ethylhexanoate (Sn(Oct)_2_), enabling the reaction to proceed efficiently at 40 °C [24]. After polymerization, the LCNCs were purified by repeated centrifugation using a chloroform/hexane cosolvent to remove the unreacted monomers and free PLLA homopolymers. Their removal was monitored by ^1^H NMR analysis of the supernatants collected during washing (Figure S1).

Figure 2a shows the FT-IR spectra of the neat CNCs and LCNCs. The neat CNCs showed a strong and broad –OH stretching band at 3300 cm^−1^, characteristic of the abundant surface hydroxyl groups. In contrast, in the spectrum of the LCNCs, the intensity of the –OH stretching band progressively decreased with increasing monomer feed ratio, and new absorption bands corresponding to the ester C=O stretching vibration of PLLA appeared at 1750 cm^−1^. These results indicate that the hydroxyl groups on the CNC surface initiated the ROP of L-lactide, leading to the grafting of PLLA onto the CNC surface. Notably, the intensity of the C=O stretching vibration peaks followed the order LCNC1 < LCNC2 < LCNC3, reflecting that the amount of grafted PLLA on the CNC surface increased in the order LCNC1 < LCNC2 < LCNC3.

To examine the grafting extent more quantitatively, ^1^H NMR analysis was performed (Figure 2b). As shown in Figure 2b, neat CNCs showed proton signals at 5.43, 5.37, and 4.32 ppm, corresponding to C2-OH, C3-OH, and C6-OH, respectively [25,26]. Interestingly, after grafting, the C6-OH signal disappeared in all LCNC samples, while the C2-OH and C3-OH peaks remained partially visible. This selective peak disappearance was attributed to the higher reactivity of the primary hydroxyl group (C6-OH) compared to that of the secondary hydroxyl groups (C2-OH, C3-OH). In addition, new peaks appeared at 5.19 ppm (peak a) and 4.19 ppm (peak a′), which were assigned to the methine (–CH) protons of PLLA, as well as at 1.42 ppm (peak b) and 1.21 ppm (peak b′), which corresponded to the methyl (–CH_3_) protons of PLLA [27,28]. These results were consistent with the FT-IR analysis and confirmed that PLLA was chemically grafted onto the CNC surface, demonstrating successful surface modification.

To quantify the amount of PLLA grafted onto the surface of the LCNCs, the DS_app_, DP, and MS_app_ values of PLLA were determined using Equations (1), (2) and (3), respectively, based on the integral ratios of the ^1^H-NMR peaks of the LCNCs in Figure 2b. The calculated values are listed in Table 1.

(1) $${\mathrm{DS}}_{\mathrm{app}}=\frac{\frac{{\mathrm{IA}}_{b^{\prime }}}{3}}{\frac{{\mathrm{IA}}_{b^{\prime }}}{3}+{\mathrm{IA}}_{\left(C2_{\_\mathrm{OH}}+C3_{\_\mathrm{OH}}+C6_{\_\mathrm{OH}}\right)}}\times 3$$

(2) $$\mathrm{DP}=\frac{\mathrm{Total}\;\mathrm{Lactyl}\;\mathrm{units}}{\mathrm{Terminal}\;\mathrm{lactyl}\;\mathrm{units}}=\frac{{\mathrm{IA}}_{\left(b+b^{\prime }\right)}}{{\mathrm{IA}}_{\left(b^{\prime }\right)}}=\frac{{\mathrm{IA}}_{\left(b\right)}}{{\mathrm{IA}}_{\left(b^{\prime }\right)}}+1$$

MS_app_ = DS_app_ × DP (3)

The DS_app_ values (1.60–1.68) were similar among the LCNC samples, whereas DP increased from 2.00 for LCNC1 to 16.1 for LCNC2 and 35.6 for LCNC3 with increasing monomer feed ratio. Accordingly, MS_app_ also increased from 3.36 to 25.6 and 56.9, respectively. Since MS_app_ reflects both DS_app_ and DP, these results are consistent with progressive PLLA graft development with increasing monomer feed ratio.

### 3.2. Crystalline Structure and Thermal Properties of LCNCs

XRD analysis was performed to observe the crystalline structures of the CNC and LCNCs (Figure 3a). As presented in Figure 3a, neat CNCs showed the characteristic diffraction peaks of cellulose I at 2θ values of 14.8° (1$\bar{1}$0), 16.5° (110), 22.4° (200), and 34.5° (400) [29,30]. The LCNCs retained the characteristic diffraction peaks of cellulose I, indicating that PLLA grafting did not alter the native crystalline structure of the CNC. However, in the diffraction pattern of LCNC3, which had the highest DP, the (1$\bar{1}$0) and (110) peaks of cellulose I were no longer distinguishable; instead, a broad diffraction halo centered at an approximate 2*θ* value of 16° appeared, corresponding to amorphous PLLA [31]. This observation suggested that the long PLLA grafts generated at the highest DP masked the diffraction from the underlying cellulose crystals.

As seen in Figure 3b, the neat CNCs showed no detectable thermal transition over the measured temperature range. In contrast, all LCNC samples showed a thermal transition corresponding to the glass transition (T_g_) of grafted PLLA: 51.5 °C (LCNC1), 57.7 °C (LCNC2), and 57.8 °C (LCNC3). This lower T_g_ of LCNC1 compared to that of LCNC2 and LCNC3 indicated insufficient DP of its grafted PLLA chains. In addition, the magnitude of the glass transition increased with increasing DP, following the order LCNC1 < LCNC2 < LCNC3.

This trend could be attributed to the greater amount of grafted PLLA with increasing DP, which resulted in a more distinct and well-defined glass transition. This increasing trend in glass transition intensity was also consistent with the increasing order of the DP (LCNC1 < LCNC2 < LCNC3) observed in the ^1^H NMR and XRD results (Figure 2b and Figure 3a). Notably, the DSC thermograms of all LCNCs exhibited only a glass transition, with no melting endotherm associated with crystalline PLLA. Since crystalline PLLA typically melts at 170–180 °C [32], the grafted PLLA chains in the LCNCs are thought to exist predominantly in an amorphous state.

To quantitatively evaluate the amount of PLLA grafted onto the CNC surface, TGA was performed on the CNCs and LCNCs (Figure 3c). The neat CNCs degraded in a single rapid step, with an onset temperature of approximately 280 °C and a peak degradation temperature of approximately 300 °C. In contrast, all LCNCs began to degrade at lower temperatures than did the neat CNCs and showed two separate degradation steps. The first degradation step, centered at approximately 200 °C, was attributed to the thermal decomposition of PLLA grafted onto the CNC surface. The second step, observed near 300 °C, was attributed to the decomposition of the CNCs. Based on the TGA curves, the first-stage weight losses of LCNC1, LCNC2, and LCNC3 were estimated to be 17.4%, 49.8%, and 64.7%, respectively. To further examine the degradation behavior, the DTG curves were also analyzed. The DTG curves (Figure 3c, inset) showed some overlap between the degradation stages, particularly for LCNC3. Therefore, these values were treated as approximate indicators of the relative PLLA-associated weight loss rather than as absolute grafted PLLA content.

### 3.3. Dispersibility of LCNCs

The dispersibility of the neat CNCs and LCNCs in DCM was investigated by visual observation, TEM, and DLS. As shown in Figure 4a, the neat CNCs showed poor dispersibility in DCM (organic solvent), forming large visible aggregates owing to the inherent hydrophilicity of the cellulose surface; this was also evident from the broad and diffuse Tyndall scattering band observed under laser illumination. In the case of LCNC1, which showed relatively low PLLA graft development, the particles were clustered into granular aggregates, similar to the case of the neat CNCs. On the other hand, LCNC2, which had a substantially greater PLLA graft development, resulted in a highly transparent suspension in DCM. This optical clarity suggested the improved dispersibility of LCNC2. Furthermore, LCNC3 exhibited a transparent and well-dispersed state with more extensive PLLA graft development. In particular, LCNC3 remained optically transparent over the concentration range of 2.5–10 mg/mL, whereas LCNC2 showed a gradual increase in turbidity in this range (Figure S2). These observations revealed that limited PLLA graft development did not effectively screen the hydrophilic cellulose surface. As MS_app_ increased, indicating greater PLLA graft development, the LCNC surface became more compatible with the organic environment, resulting in improved dispersibility. Thus, higher MS_app_ values were associated with improve dispersibility and greater compatibility with hydrophobic polymer matrices.

The results of TEM analysis further supported these findings. As shown in Figure 4a, the neat CNCs formed large aggregated domains in the organic solvent phase. LCNC1 showed slightly reduced aggregation compared to the neat CNCs but still formed noticeable clusters. In contrast, both LCNC2 and LCNC3, which had higher MS_app_ values than LCNC1, showed high dispersibility without significant aggregation. DLS measurements (Figure 4b) showed hydrodynamic size distributions ranging from approximately 100 to 900 nm for LCNC2 and LCNC3 in DCM, indicating reduced aggregation compared with CNC and LCNC1.

### 3.4. Optical and Mechanical Properties of PLA/LCNC Nanocomposites

Figure 5a shows the photographs of the PLA nanocomposite films reinforced with 1 wt% neat CNCs and LCNCs. As discussed in Section 3.3, the PLA/CNC nanocomposite film had the highest opacity due to the poor dispersion of CNCs within the PLA matrix. The PLA/LCNC1 nanocomposite film was also found to be opaque. Even the PLA/LCNC2 nanocomposite film, which was better dispersed in the PLA, exhibited slight turbidity. On the other hand, the PLA/LCNC3 nanocomposite film comprising LCNC3 with the highest DP and superior dispersion maintained excellent optical clarity. UV–vis spectroscopy showed that the transmittance of PLA/LCNC3 in the visible region was nearly identical to that of neat PLA (Figure 5b). A similar trend was also observed for the solution-cast films prior to hot-pressing (Figure S3). These results indicate that increasing PLLA graft development, accompanied by increasing graft-chain DP, is associated with improved LCNC dispersion and high optical transparency in the PLA nanocomposites.

The mechanical properties of the PLA nanocomposites reinforced with CNCs and LCNCs at a fixed total filler loading of 1 wt% are presented in Figure 6 and Table 2. Although the actual CNC content may differ among the LCNC samples, the results are discussed by comparing the different PLLA-functionalized CNC formulations at the same total filler loading. The stress–strain curves (Figure 6a) reveal distinct differences in mechanical behavior depending on the filler type, with more extensive PLLA graft development being associated with improved mechanical properties of the PLA/LCNC nanocomposites. As shown in Figure 6b, neat PLA had a tensile strength of 60.6 ± 1.39 MPa and showed typical brittle fracture behavior. The PLA/CNC nanocomposite had nearly identical tensile strength (60.8 ± 2.06 MPa), indicating that the incorporation of unmodified CNCs had a negligible effect on the tensile strength. The PLA/LCNC1 nanocomposite also possessed similar mechanical strength (61.3 ± 0.45 MPa) to PLA/CNC. The similarity in tensile strength observed for the PLA/CNC and PLA/LCNC1 nanocomposites was attributable to the aggregation of the CNCs and LCNC1 fillers resulting from their poor dispersion in the organic phase, as discussed above. However, the tensile strength increased to 64.5 ± 1.89 MPa for PLA/LCNC2. Furthermore, the PLA/LCNC3 nanocomposite, which contained LCNC3 with the highest MS_app_, showed a slightly higher mean tensile strength than PLA/LCNC2, reaching 66.9 ± 0.49 MPa. Considering that PLA, the PLA/CNC nanocomposite, and the PLA/LCNCs nanocomposites were amorphous (Figure S4), the enhanced mechanical strength observed for PLA/LCNC3 was solely due to the reinforcing effect of the fillers rather than the crystallization of the PLA matrix.

To further examine the filler distribution in the PLA nanocomposites, the tensile fracture surfaces of neat PLA, PLA/LCNC1, and PLA/LCNC3 were observed by FE-SEM (Figure 7). Neat PLA showed a relatively smooth fracture surface. In contrast, several aggregates were observed in PLA/LCNC1, indicating poor dispersion in the PLA matrix. However, PLA/LCNC3 showed a relatively smooth and uniform fracture surface similar to that of neat PLA, suggesting improved dispersion of LCNC3 in the PLA matrix. This improved dispersion may allow more effective stress transfer between LCNC3 and the PLA matrix. This observation is consistent with the tensile test results, where PLA/LCNC3 exhibited higher tensile strength than neat PLA and PLA/LCNC1. Taken together, FT-IR and ^1^H NMR confirmed PLLA grafting, XRD showed preservation of CNC crystallinity, and TEM/FE-SEM revealed improved filler dispersion, collectively supporting the enhanced mechanical performance of the PLA/LCNC nanocomposites.

To further investigate the reinforcing effect of the LCNCs, the dynamic mechanical behavior and stress relaxation behavior of PLA, the PLA/CNC nanocomposite, and the PLA/LCNC nanocomposites were examined by DMA (Figure 8). As shown in Figure 8a, the onset temperature at which the storage modulus began to decrease was 67.6 °C for neat PLA and 71.2 °C, 72.1 °C, 73.3 °C, and 76.3 °C for PLA/CNC, PLA/LCNC1, PLA/LCNC2, and PLA/LCNC3, respectively. The onset temperature reflects the energy required for the PLA chain segments to gain mobility; therefore, a higher onset temperature indicates greater restriction of chain mobility. Notably, PLA/LCNC3 showed the highest onset temperature among the samples. This result suggests that the improved compatibility between LCNC3 and the PLA matrix imposed greater constraints on the segmental mobility of the surrounding PLA chains. In addition, the rubbery plateau observed for PLA/LCNC3 spanned a wider temperature range than that in the case of the other samples. This behavior may be associated with enhanced interfacial interactions and restricted PLA chain mobility, with a possible contribution from physical entanglement between the grafted PLLA and PLA chains.

The stress relaxation behavior followed a similar trend to the dynamic mechanical behavior (Figure 8b). Neat PLA underwent severe relaxation, with the modulus decreasing by 43.9% over 500 s. The incorporation of unmodified CNCs partially suppressed this decline (28.4% decrease), and LCNC1 further improved the relaxation resistance (19.1% decrease). In particular, PLA/LCNC2 and PLA/LCNC3 showed superior resistance to stress relaxation, with only 15.2% and 12.4% decreases in the modulus, respectively. These results suggest that the increased PLLA grafting of LCNCs improved their interaction with the PLA matrix and restricted the mobility of the surrounding PLA chains. This enhanced interfacial interaction may contribute to the improved mechanical properties of the PLA/LCNC nanocomposites. Therefore, the progressive development of PLLA grafts, accompanied by increasing graft-chain DP, is associated with enhanced reinforcing efficiency of LCNCs in PLA nanocomposites (Figure 9).

## 4. Conclusions

In this study, poly(L-lactic acid) (PLLA)-grafted CNCs (LCNCs) with progressive PLLA graft development, accompanied by increasing graft-chain degree of polymerization (DP), were successfully prepared while maintaining similar apparent degree of substitution (DS_app_) values. Fourier transform infrared (FT-IR) and ^1^H nuclear magnetic resonance (^1^H NMR) analyses confirmed successful PLLA grafting, and X-ray diffraction (XRD) patterns showed that the intrinsic crystallinity of the CNCs was largely preserved after modification. Dispersion studies in dichloromethane (DCM) showed that LCNC3 (apparent molar substitution, MS_app_ = 56.9) exhibited improved dispersibility, whereas LCNC1 (MS_app_ = 3.36) remained strongly aggregated. These results indicate that progressive PLLA graft development, reflected by higher MS_app_ values, was associated with improved LCNC dispersibility. FE-SEM observations of the tensile fracture surfaces further showed a more uniform morphology for PLA/LCNC3 than for PLA/LCNC1, supporting improved filler dispersion in the final nanocomposite. This enhanced dispersion enabled PLA/LCNC3 to retain optical transparency comparable to that of neat PLA while exhibiting significantly higher tensile strength than PLA/LCNC1. Overall, progressive PLLA graft development, reflected by increasing MS_app_, was associated with improved dispersion, optical transparency, and mechanical properties of the PLA nanocomposites. These findings suggest that MS_app_ can serve as a useful parameter for describing the PLLA grafting level in the design of high-performance, optically transparent PLA-based materials.

## Figures and Tables

**Figure 1:**
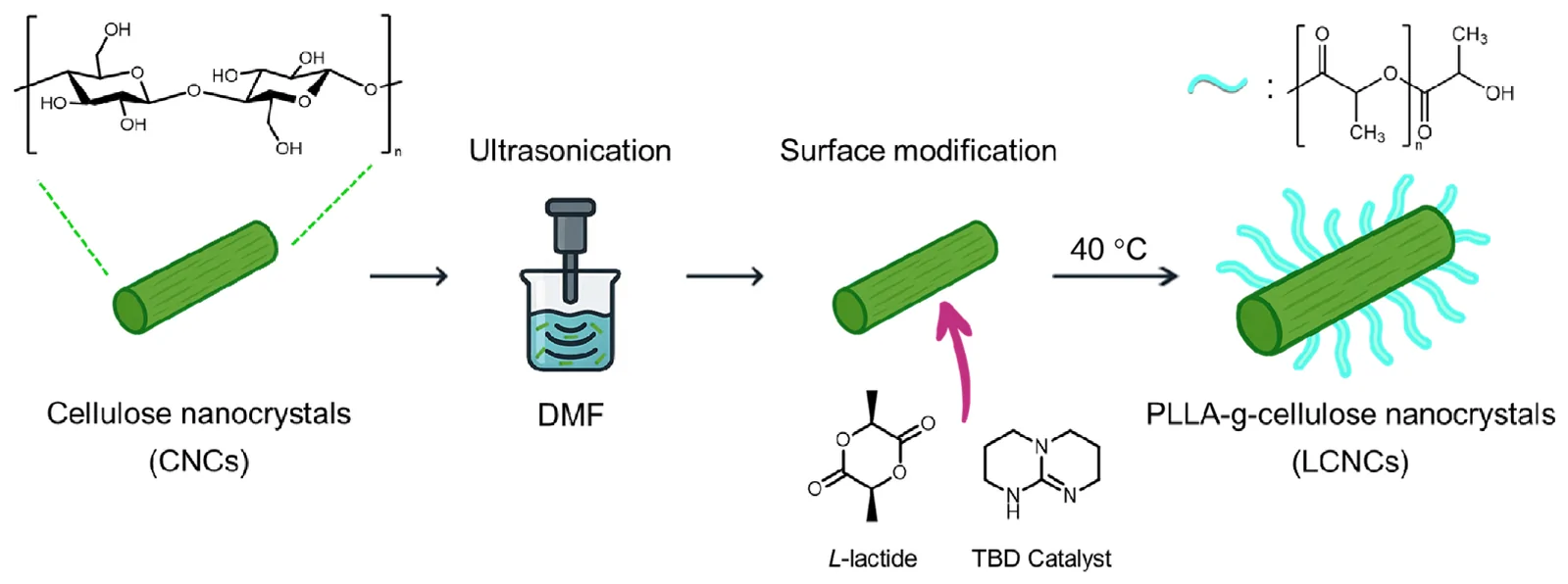
Schematic illustration of the synthesis of PLLA-g-CNCs (LCNCs).

**Figure 2:**
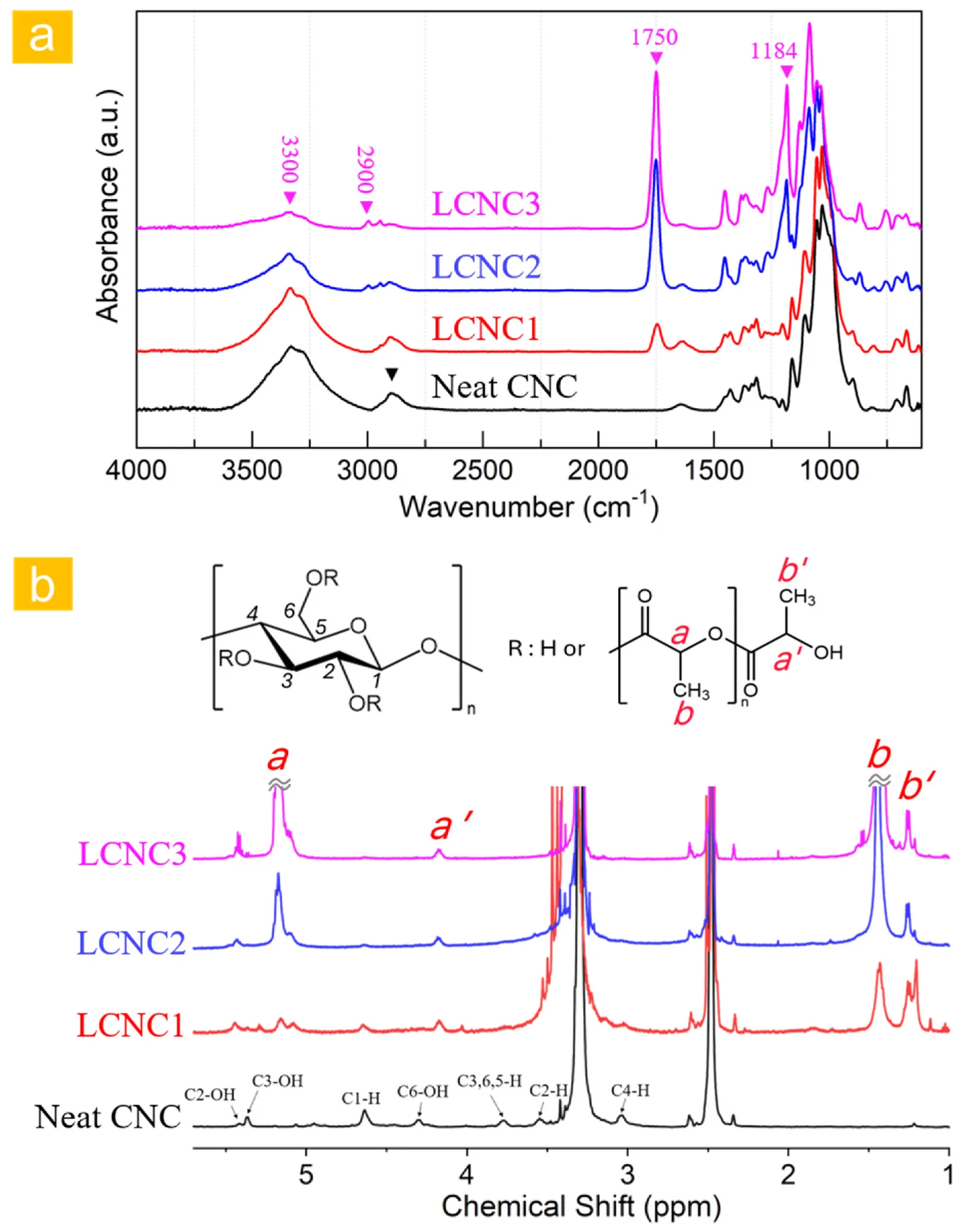
(**a**) FT-IR and (**b**) ^1^H-NMR spectra of neat CNCs and LCNCs.

**Figure 3:**
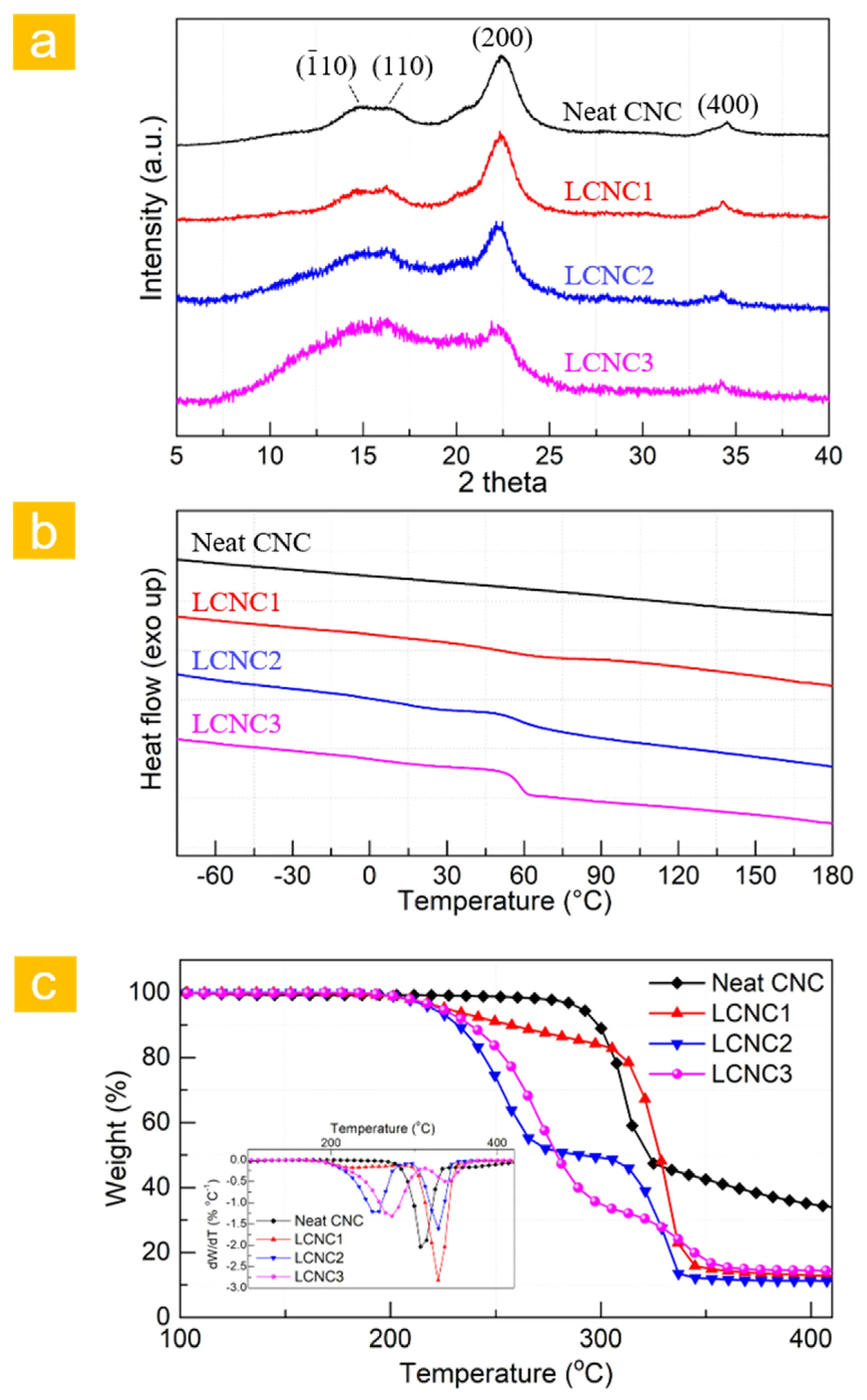
(**a**) XRD patterns, (**b**) DSC thermograms, and (**c**) TGA curves with DTG curves (**inset**) of CNCs and LCNCs.

**Figure 4:**
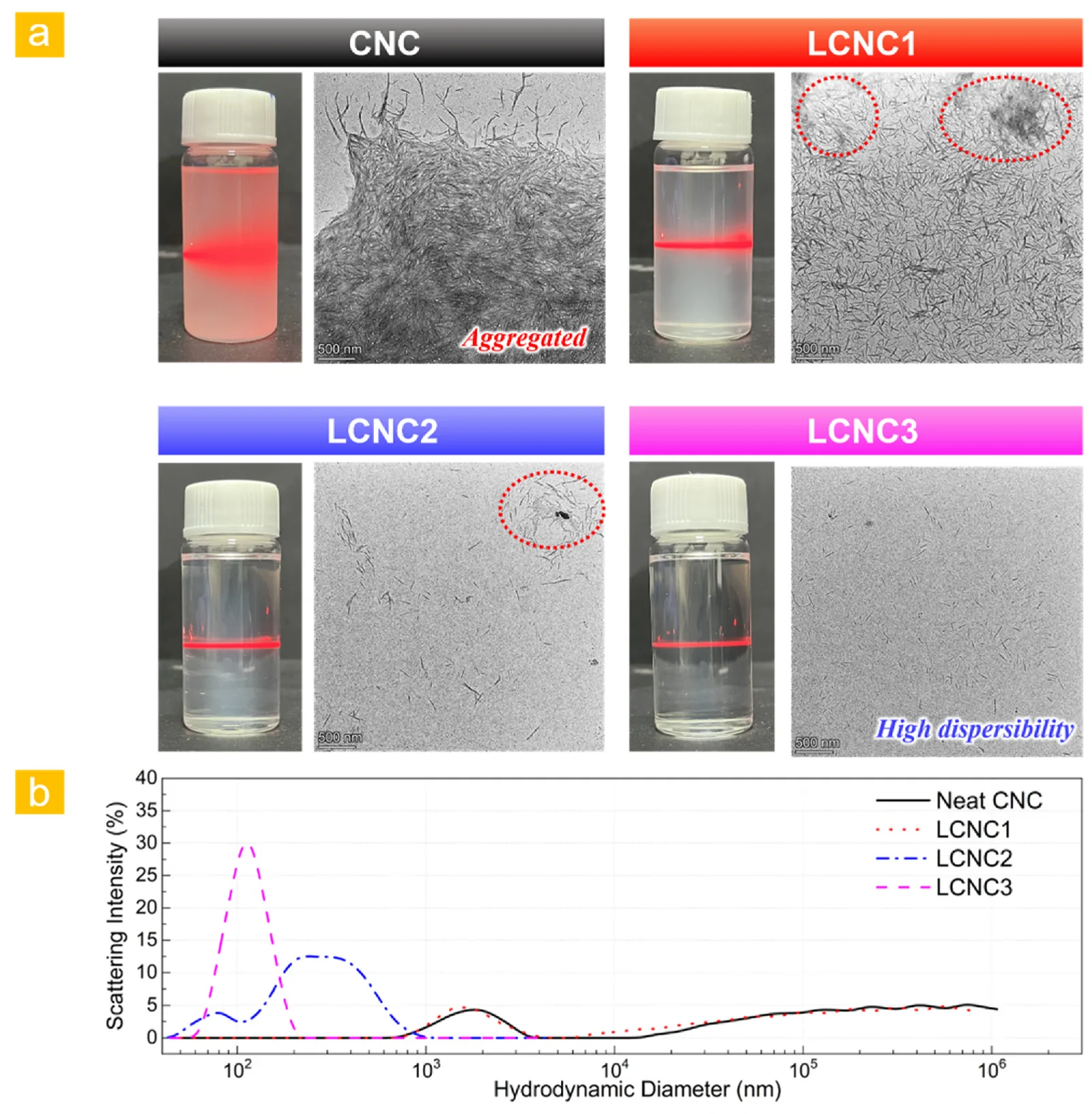
(**a**) Visual dispersion state and TEM images of neat CNCs and LCNCs in DCM. The red dotted circles indicate aggregated regions observed in the TEM images. (**b**) Hydrodynamic diameter distributions of neat CNCs, LCNC1, LCNC2, and LCNC3, as measured by DLS.

**Figure 5:**
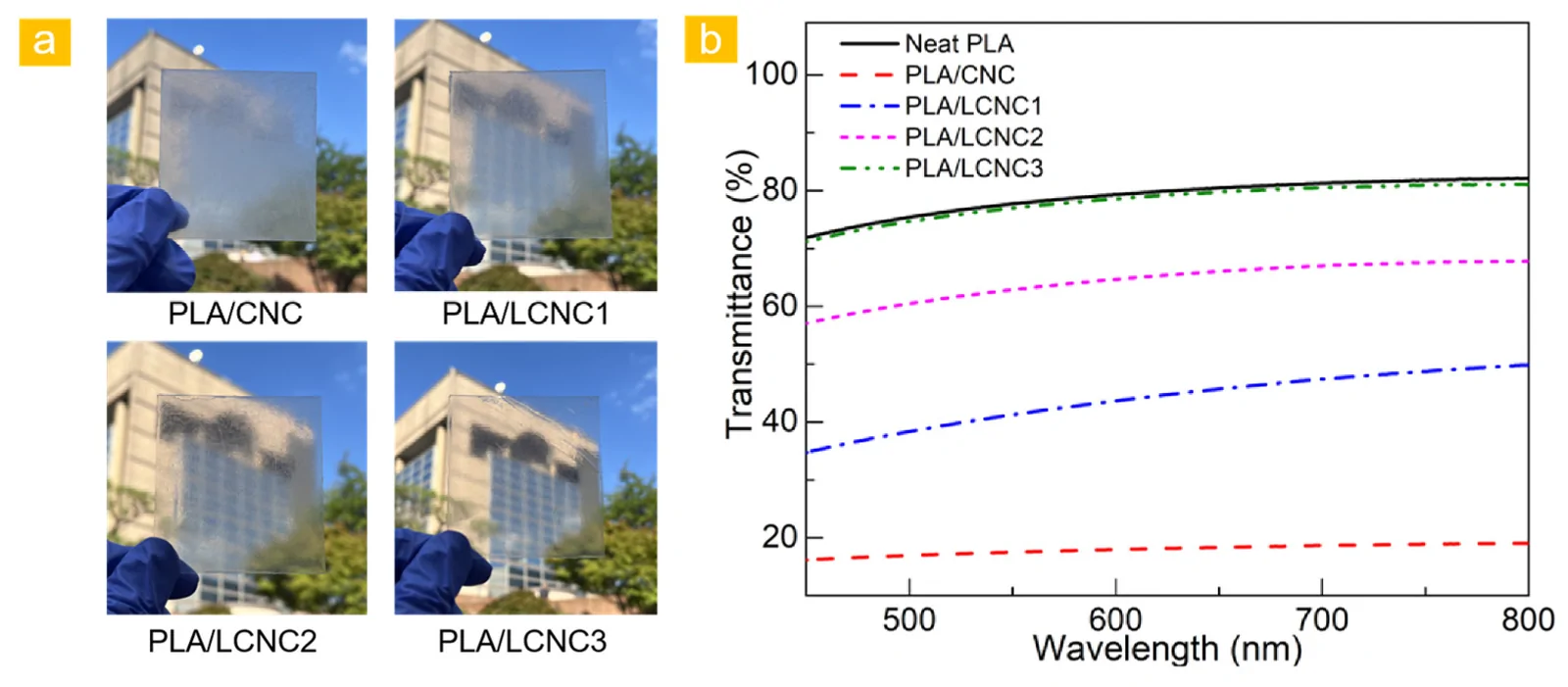
Optical transparency of PLA nanocomposites incorporating 1.0 wt% CNCs and LCNCs. (**a**) Photographs of hot-pressed films and (**b**) UV–Vis transmittance spectra.

**Figure 6:**
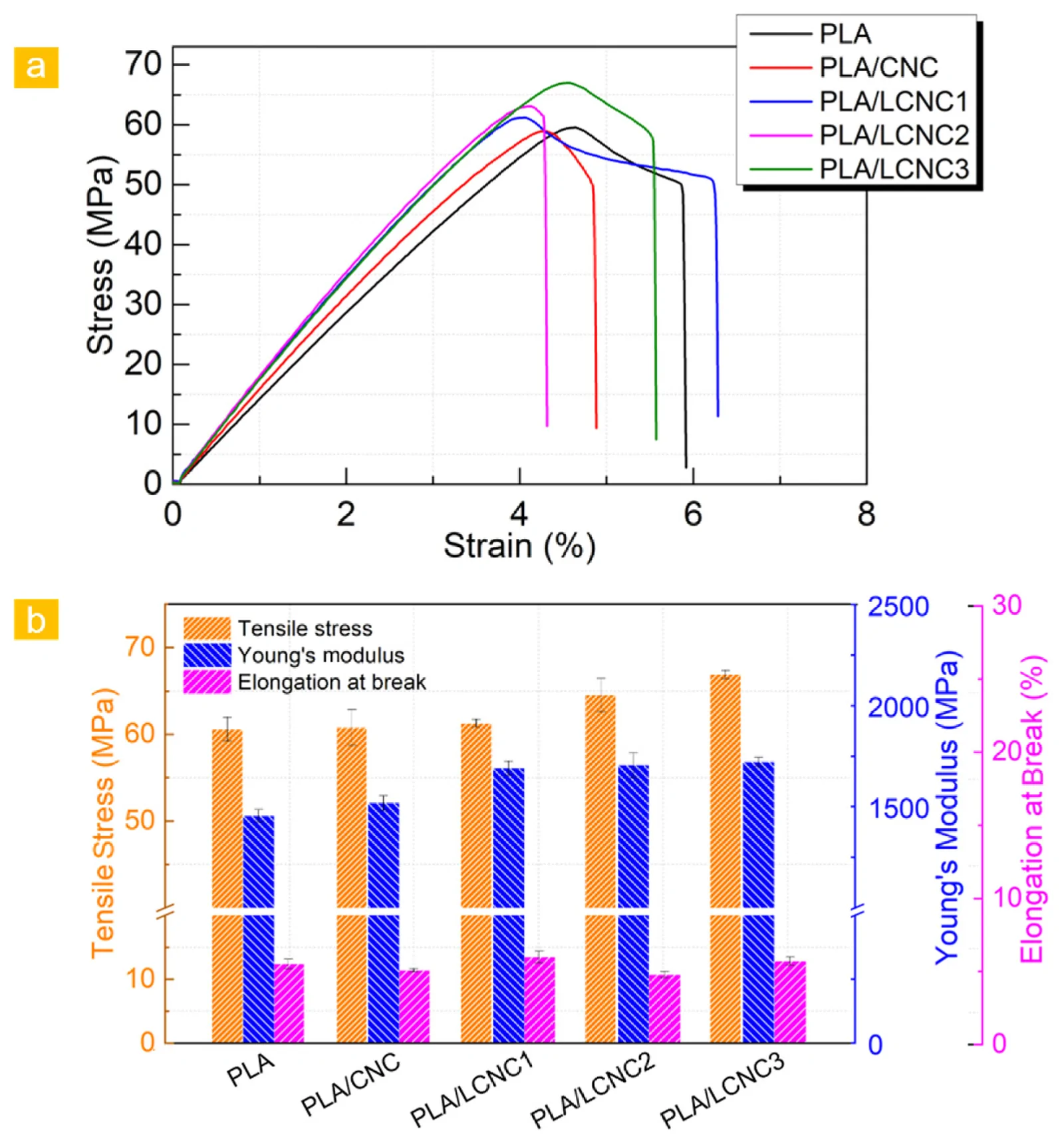
Tensile properties of PLA, PLA/CNC, and PLA/LCNC nanocomposites containing 1.0 wt% filler: (**a**) representative stress–strain curves and (**b**) tensile strength, elongation at break, and Young’s modulus.

**Figure 7:**
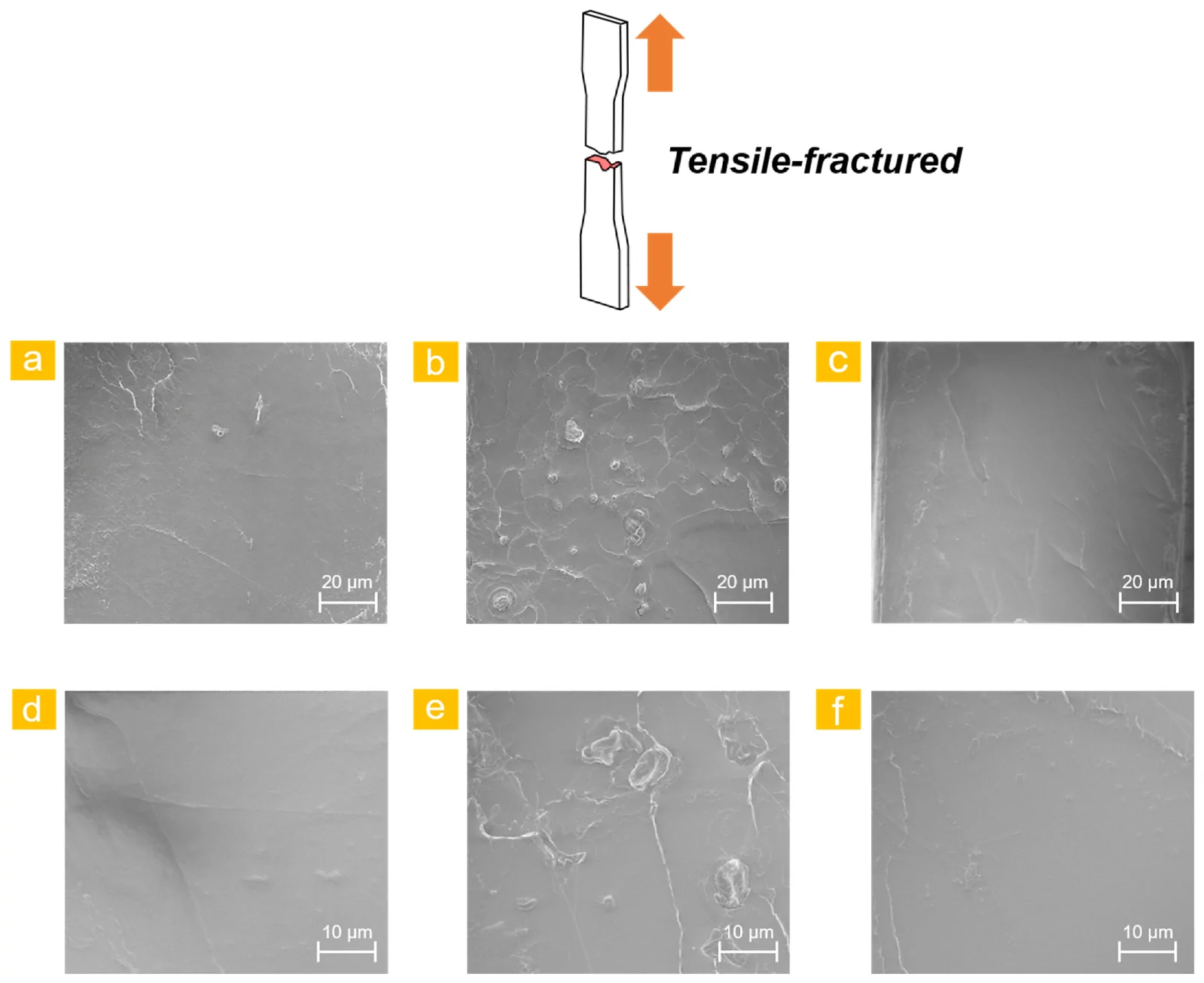
FE-SEM images of the tensile fracture surfaces of (**a**,**d**) neat PLA, (**b**,**e**) PLA/LCNC1, and (**c**,**f**) PLA/LCNC3 at low (**a**–**c**) and high (**d**–**f**) magnifications.

**Figure 8:**
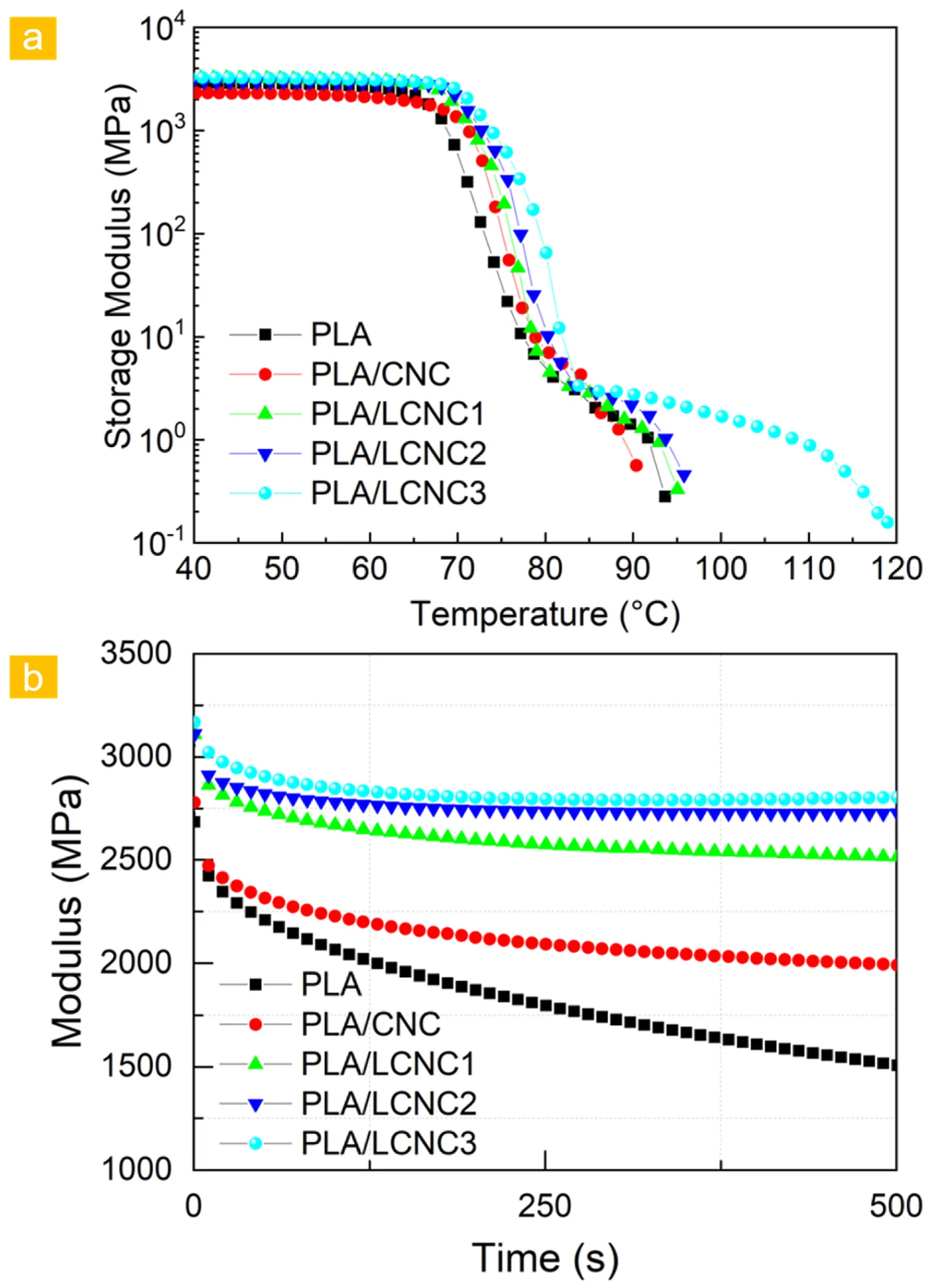
Dynamic mechanical properties of neat PLA and PLA nanocomposites containing 1 wt% CNCs and LCNCs: (**a**) Storage modulus as a function of temperature and (**b**) stress relaxation behavior measured at 40 °C under 0.1% strain.

**Figure 9:**
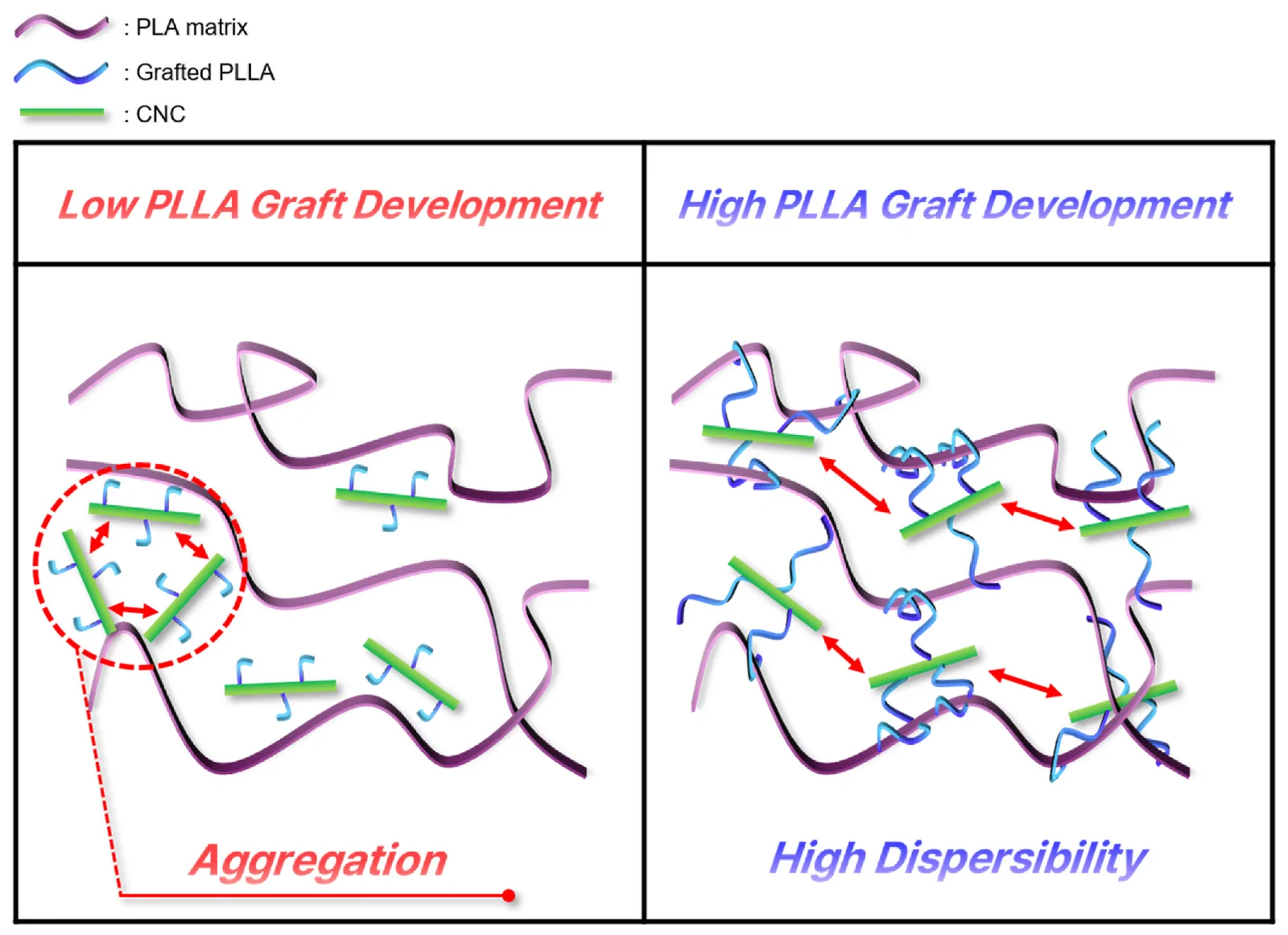
Schematic illustration of the effect of PLLA graft development on LCNC dispersion in the PLA matrix, showing aggregation at low grafting extent and improved dispersion at high grafting extent.

**Table 1:** Apparent degree of substitution (DS_app_), degree of polymerization (DP), and apparent molar substitution (MS_app_) of LCNCs.

Sample Code	Lactide/CNC Feed Ratio (g/g)	DS_app_ ^1^	DP ^2^	MS_app_ ^3^
LCNC1	1	1.68	2.00	3.36
LCNC2	5	1.60	16.1	25.6
LCNC3	10	1.60	35.6	56.9

^1^ Calculated using Equation (1). ^2^ Calculated using Equation (2). ^3^ Calculated using Equation (3).

**Table 2:** Mechanical properties of neat PLA and PLA nanocomposites with CNC and LCNC fillers (1 wt%).

Sample Code	Tensile Strength (MPa)	Young’s Modulus (MPa)	Elongation at Break (%)
PLA	60.6 ± 1.39 ^a,^*	1455 ± 29.8 ^b^	5.42 ± 0.33 ^ab^
PLA/CNC	60.8 ± 2.06 ^a^	1519 ± 36.3 ^b^	4.98 ± 0.11 ^bc^
PLA/LCNC1	61.3 ± 0.45 ^a^	1690 ± 34.8 ^a^	5.88 ± 0.39 ^a^
PLA/LCNC2	64.5 ± 1.89 ^b^	1705 ± 61.6 ^a^	4.69 ± 0.22 ^c^
PLA/LCNC3	66.9 ± 0.49 ^b^	1721 ± 23.1 ^a^	5.61 ± 0.28 ^a^

* Different superscript letters within the same column indicate statistically significant differences (*p* < 0.05), as determined by one-way ANOVA followed by Tukey’s post hoc test.

## Data Availability

The original contributions presented in the study are included in the article. Further inquiries can be directed to the corresponding author.

## Supplementary Materials

The following supporting information can be downloaded at https://www.mdpi.com/article/10.3390/polym18192370/s1, Figure S1. ^1^H-NMR spectra of supernatants after 2, 3, and 4 washing cycles showing progressive removal of free PLLA chains; Figure S2. Optical appearance of LCNC2 and LCNC3 dispersions in DCM at concentrations ranging from 2.5 to 10 mg/mL; Figure S3. Optical transparency of PLA nanocomposites incorporating CNC and LCNCs: (a) Photographs of solution-cast films and (b) UV–Vis transmittance spectra; Figure S4. DSC thermograms of neat PLA and PLA nanocomposites with CNCs and LCNCs (1.0 wt%).

## Abbreviations

The following abbreviations are used in this manuscript:

PLA	Poly(lactic acid)
PLLA	Poly(L-lactic acid)
CNC(s)	Cellulose nanocrystal(s)
LCNC(s)	PLLA-grafted cellulose nanocrystal(s)
DS_app_	Apparent degree of substitution
DP	Degree of polymerization
MS_app_	Apparent molar substitution
ROP	Ring-opening polymerization
DMF	N,N-Dimethylformamide
DCM	Dichloromethane
TBD	1,5,7-Triazabicyclo[4.4.0]dec-5-ene
DMAP	4-Dimethylaminopyridine
Sn(Oct)_2_	Tin(II) 2-ethylhexanoate
