# Supplementary material for: Effect of Graft Development in PLLA-Functionalized Cellulose Nanocrystals on Dispersibility, Transparency, and Mechanical Reinforcement in PLA Nanocomposites
Source: Polymers (Basel). 2026 Sep 29;18(19):2370. doi: 10.3390/polym18192370 (PMC13666614; doi:10.3390/polym18192370)
Supplement: Supplementary file 1 [file polymers-18-02370-s001.zip › polymers-4574041-supplementary.pdf]

Supporting Information

Effect of Graft Development in PLLA-Functionalized Cellulose Nanocrystals on Dispersibility, Transparency, and Mechanical Reinforcement in PLA Nanocomposites

Hee Cheol Kang and Jae Woo Chung *

Department of Materials Science and Engineering, Soongsil University, 369 Sangdo-ro, Dongjak-gu, Seoul 06978, Republic of Korea; wkdehrgml@gmail.com
* Correspondence: jwchung@ssu.ac.kr; Tel.: +82-2-828-7047

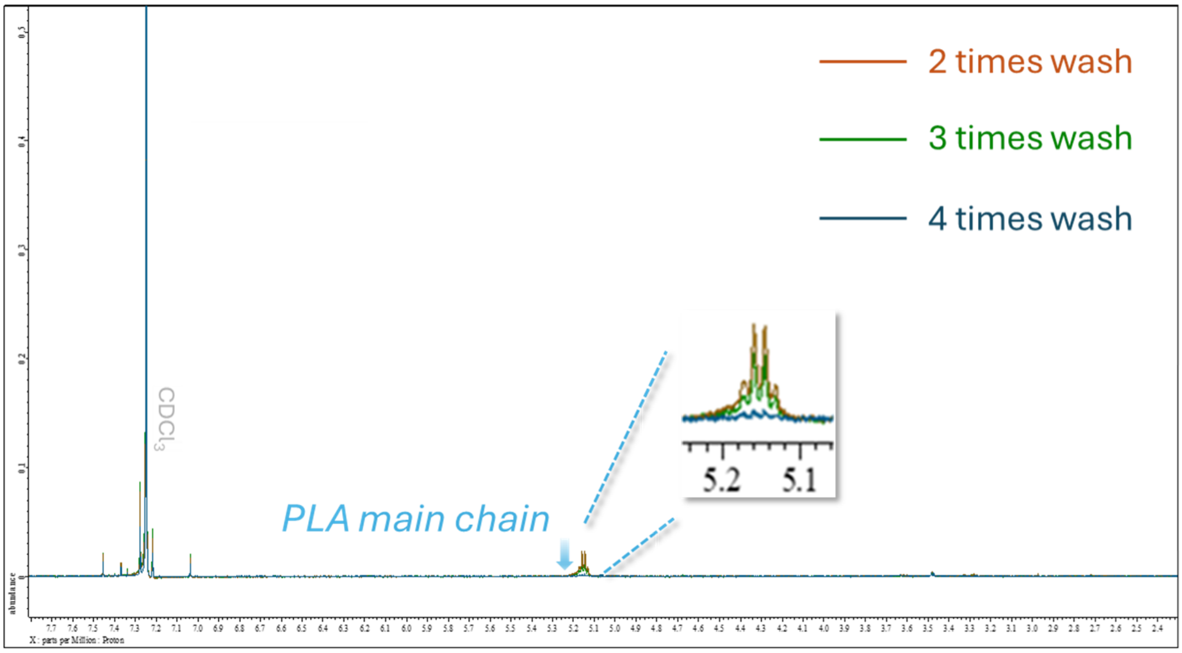

Figure S1. ^{1}H-NMR spectra of supernatants after 2, 3, and 4 washing cycles showing progressive removal of free PLLA chains.

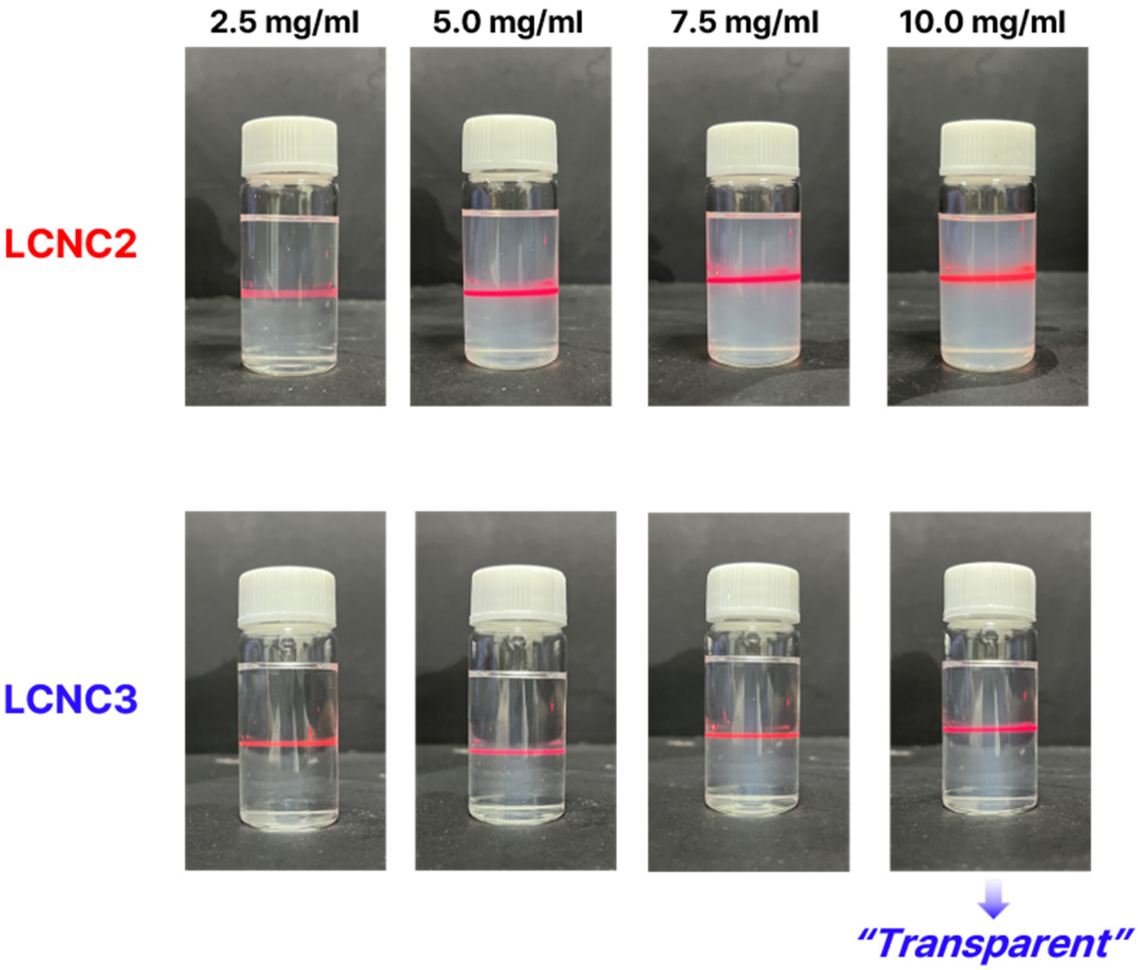

Figure S2. Optical appearance of LCNC2 and LCNC3 dispersions in DCM at concentrations ranging from 2.5 to 10 mg/mL.

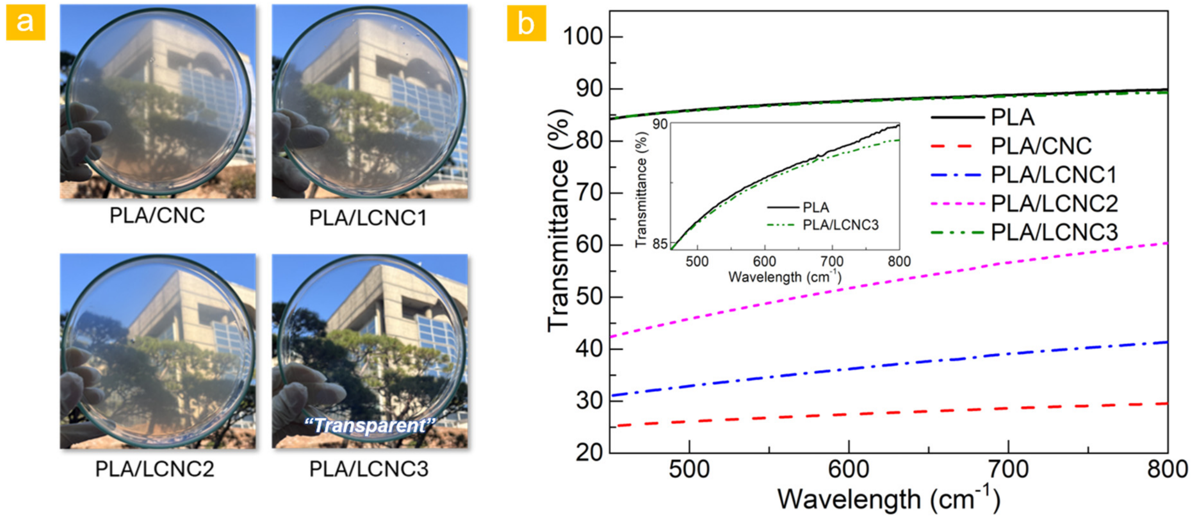

Figure S3. Optical transparency of PLA nanocomposites incorporating CNC and LCNCs. (a) Photographs of solution-cast films and (b) UV–Vis transmittance spectra

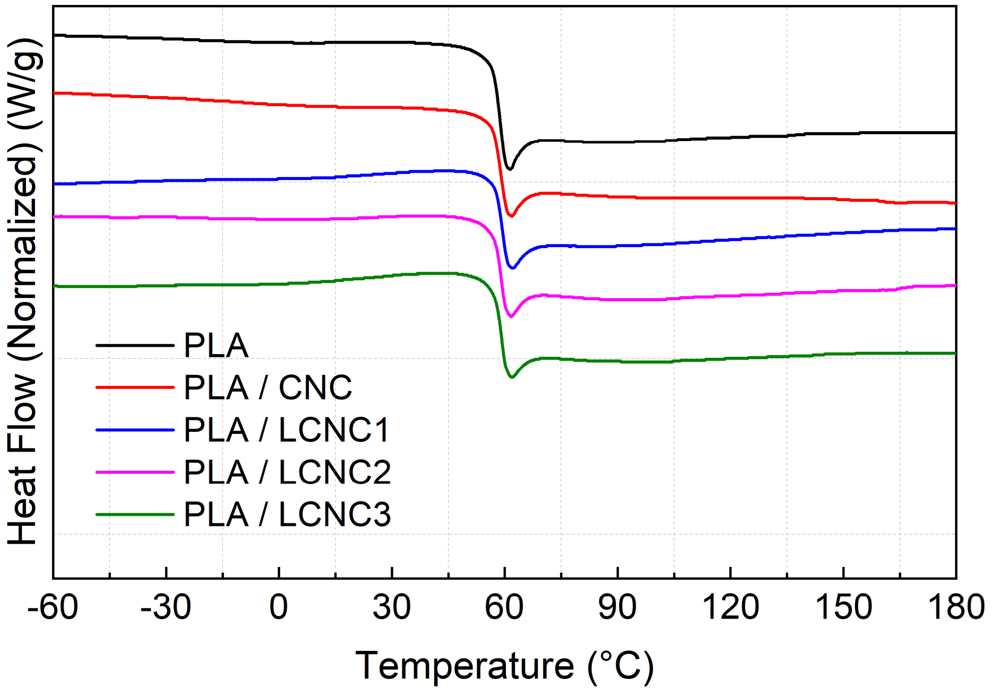

Figure S4. DSC thermograms of neat PLA and PLA nanocomposites with CNCs and LCNCs (1.0 wt%).
